# The Molecular Basis of Organic Chemiluminescence

**DOI:** 10.3390/bios13040452

**Published:** 2023-04-03

**Authors:** Maidileyvis C. Cabello, Fernando H. Bartoloni, Erick L. Bastos, Wilhelm J. Baader

**Affiliations:** 1 Departamento de Química Fundamental, Instituto de Química, Universidade de São Paulo, Av. Prof. Lineu Prestes 748, São Paulo 05508-000, Brazil; 2 Centro de Ciências Naturais e Humanas, Universidade Federal do ABC, Avenida dos Estados 5001, Santo André 09210-580, Brazil

**Keywords:** chemiluminescence, bioluminescence, cyclic peroxides, 1,2-dioxetanes, 1,2-dioxetanones, sensors, ultrasensitive detection

## Abstract

Bioluminescence (BL) and chemiluminescence (CL) are interesting and intriguing phenomena that involve the emission of visible light as a consequence of chemical reactions. The mechanistic basis of BL and CL has been investigated in detail since the 1960s, when the synthesis of several models of cyclic peroxides enabled mechanistic studies on the CL transformations, which led to the formulation of general chemiexcitation mechanisms operating in BL and CL. This review describes these general chemiexcitation mechanisms—the unimolecular decomposition of cyclic peroxides and peroxide decomposition catalyzed by electron/charge transfer from an external (intermolecular) or an internal (intramolecular) electron donor—and discusses recent insights from experimental and theoretical investigation. Additionally, some recent representative examples of chemiluminescence assays are given.

## 1. Fundamental Aspects of Organic Chemiluminescence

Luminescence is a broad term used to describe the spontaneous emission of radiation from an electronically or vibrationally excited species. Fluorescence and phosphorescence are common forms of luminescence that are observable when visible light is emitted. In these processes, the energy used to produce the excited state comes from the photons that are absorbed by the molecular entity. Phenomenologically, phosphorescence is long-term luminescence, while fluorescence is only observable during irradiation. This is because phosphorescence involves a change in the electron spin multiplicity that, for organic compounds, often involves intersystem crossing between singlet and triplet excited states. Chemiluminescence (CL) and bioluminescence (BL) are forms of luminescence fueled by chemical energy instead of light absorption. In a chemiluminescent reaction, the decomposition of suitable high-energy intermediates (HEIs) generates products in their electronically excited states. Bioluminescence results from metabolic transformations in which HEIs are formed. In both processes, singlet and triplet excited species can be produced, and when their decay to the ground state is followed by visible luminescence, the viewer can experiment with one of the most intriguing and interesting natural phenomena.

**Scheme 1:**
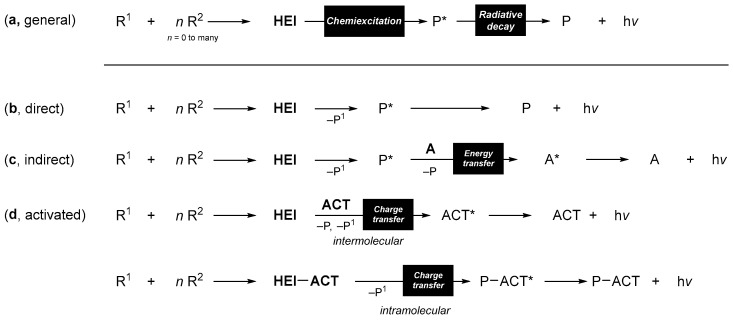
Mechanisms in Organic Chemiluminescence. (**a**) General mechanism. (**b**) Direct chemiluminescence transformation. (**c**) Indirect CL in the presence of a fluorescent/phosphorescent energy acceptor. (**d**) Activated CL in the presence of an oxidizable fluorescent dye. R—reagents; P—products; HEI—high-energy intermediate; P*—excited state product; A—electronic energy acceptor; A*—excited state acceptor; ACT—activator; ACT*—excited state activator; HEI-ACT—activator linked to high-energy intermediate; P-ACT—ACT linked to the product; P-ACT*—excited state ACT linked to the product.

The occurrence and characteristics of chemiluminescence of organic compounds in solution, such as emission wavelength, intensity, and rate, are affected by external agents and, hence, have found numerous analytical and bioanalytical applications [1]. In one or several elementary reaction steps, two or more reagents can produce the HEI. The decomposition of this intermediate, giving a triplet and/or singlet excited species, is called chemiexcitation, a process that may occur directly or via intra- or intermolecular electron/charge transfer (Scheme 1a). The fate of the excited species depends mostly on its structural and electronic properties as well as the surrounding microenvironment.

Highly fluorescent or phosphorescent compounds produced by direct chemiexcitation can undergo *direct* chemiluminescence (Scheme 1b). However, the decay of emissive singlet- and triplet-excited species concurs with energy transfer to suitable acceptors. As a result, singlet-excited species can transfer energy to fluorescent dyes, enabling the modulation of color and intensity of emission in so-called *indirect* chemiluminescence (Scheme 1c). Nevertheless, when triplet-excited species are formed, energy transfer to dioxygen (^3^O_2_) quenches emission and may result in photosensitization (although the use of chemiluminescence to produce singlet oxygen is still elusive). Hence, oxygen must be removed from the solution to maximize emission, and singlet-excited species are much more common in chemiluminescent reactions than their triplet counterparts. Some organic compounds display activated (or catalyzed/sensitized) chemiluminescence, a process in which a chemical reaction between the HEI and an oxidizable fluorescent compound (namely, the activator, ACT) leads to the chemiexcitation of the ACT (Scheme 1d). In some chemiluminescent reactions, the ACT is part of the reagent in a triggered form, making light emission controllable by using deprotecting agents.

Measuring chemiluminescence requires, in principle, only a photon detector. The chemiluminescence spectrum (CL intensity vs. wavelength) is usually acquired using a fluorescence spectrometer with the lamp turned off, whereas kinetic data can be obtained using simple luminometers, which, despite being far more sensitive, commonly lack spectral resolution. Many direct and indirect chemiluminescent reactions are initiated by increasing the temperature of the solution to a point where the HEI undergoes thermal decomposition, which can be as low as 25 °C or as high as 300 °C depending on the chemical system. However, most activated reactions occur at room temperature or lower upon the addition of an activator or a chemical or enzymatic agent to deprotect the activator and, henceforth, are more convenient for the development of analytical assays. As soon as the reaction begins to produce light, the detector system accumulates the signal for a defined period. As the detection and optical systems vary according to the equipment used, the amount of light produced cannot be accessed directly. Despite this, the emission intensity (originally in relative light units/time or arbitrary units (a.u.)/time) is proportional to the concentration of the limiting reagents at any point of the reaction and, hence, the area under the kinetic curve is proportional to the total number of photons emitted [2,3].

If the number of photons reaching the detector is known, the emission intensity can be reported as moles of photons (or Einsteins, E, a non-SI unit) per time unit, typically seconds, and is proportional to the reaction rate. When a calibrated measuring system is not available, the use of the luminol reaction under standard conditions provides a convenient way to know how many photons are reaching the detection unit under a specific experimental setup. Because luminol emits blue light, calibration may require additional correction for changes in the sensitivity of the detector with the emission wavelength. When the emission intensity is converted from a.u. s^–1^ to E s^–1^, integration of the kinetic curve (extrapolated to infinity over time in s) gives the number of moles of photons emitted. Dividing this quantity by the amount of limiting reagent gives the chemiluminescence quantum yield (*Φ*_CL_, dimensionless), which is a figure of merit representing the efficiency of a chemiluminescent reaction (Equation (1)) [1,4,5].

The *Φ*_CL_ also represents the product of the chemiexcitation quantum yield (^CE^*Φ*, either singlet quantum yield,^1^*Φ*, or triplet quantum yield, ^3^*Φ*) and the emission quantum yield (*Φ*_EM_, either fluorescence quantum yield, *Φ*_FL_, or phosphorescence quantum yield, *Φ*_P_) of the emitting species. Therefore, for most chemiluminescent reactions based on organic compounds, knowing the *Φ*_CL_ of the system and the *Φ*_FL_ of the emitting species enables the determination of the *^1^Φ*, which represents the efficiency of the chemiexcitation step (Equation (2)) [3,4]. Conventional kinetic measurements and the determination of reaction rate constants enable the study of the steps preceding the formation of the HEI and, therefore, do not give kinetic insight into chemiexcitation. However, together with quantum yields, the analysis of these parameters is the foundation of the mechanistic study of chemiluminescent reactions and, ultimately, provides a rationale for how they work and the limitations of their use in analytical methods [5].

*Φ*_CL_ = photons emitted/molecules of limiting CL reagent = ^CE^*Φ* × *Φ*_EM_ (1)

^1^*Φ* = *Φ*_CL_/*Φ*_FL_ (2)

Despite this formalism, the meaning of CL efficiency can be easily misinterpreted. For example, the chemiluminescence of luminol in an aqueous alkaline medium (pH around 11) is frequently used for demonstration purposes due to its intense and visually appealing blue glow; however, the *Φ*_CL_ of this reaction is approximately 1%, implicating that large amounts of luminol are required to obtain the desired effect. Efficient reactions are expected to have chemiluminescence quantum yields of at least 10%, and the most important examples are the triggered decomposition of 1,2-dioxetanes and the oxidation of oxalic esters by hydrogen peroxide in the presence of an ACT (peroxyoxalate reaction/system), which occur via intramolecular and intermolecular activated chemiluminescence, respectively. The bioluminescence of the firefly luciferin is another example of efficient (*Φ*_BL_ = 40%) intramolecular activated transformation, which requires enzymatic activation to trigger the emission [6]. Activated chemiluminescence is generally more efficient than direct chemiluminescence; however, not all activated reactions are efficient. The following discussion will elaborate on each of these mechanisms and compare the efficiency and application of notable CL reactions.

## 2. General Chemiexcitation Mechanisms

**Scheme 2:**
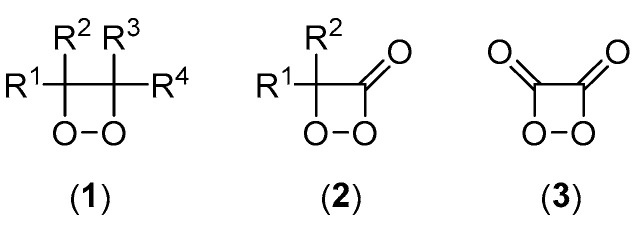
Structures of important high-energy intermediates participating in chemiluminescent reactions. The R groups are alkyl, aryl, and, eventually, H.

Strained cyclic peroxides are the high-energy intermediates of many efficient CL and BL transformations. Therefore, modern studies on the mechanisms of chemiexcitation are intimately linked to the synthesis of such peroxides, more specifically, 1,2-dioxetanes (**1**) and 1,2-dioxetanones (**2**) (Scheme 2) [7]. The first synthesis of a 1,2-dioxetane derivative, 3,3,4-trimethyl-1,2-dioxetane, was reported by Kopecky and Mumford [8], whereas Adam and Liu described the first synthesis of a 1,2-dioxetanone derivative, 3-*tert*-butyl-1,2-dioxetanone [9]. The extremely unstable unsubstituted 1,2-dioxetane has been synthesized and characterized by Adam and Baader [10]; however, the unsubstituted 1,2-dioxetanone has never been obtained, supposedly due to its higher instability. The last member of the trinity of four-membered ring peroxides essential for CL is the elusive 1,2-dioxetanedione (**3**), which was postulated long ago as the HEI in the peroxyoxalate reaction [11,12,13,14], a hypothesis that has been supported by recently available experimental data [15].

**Scheme 3:**
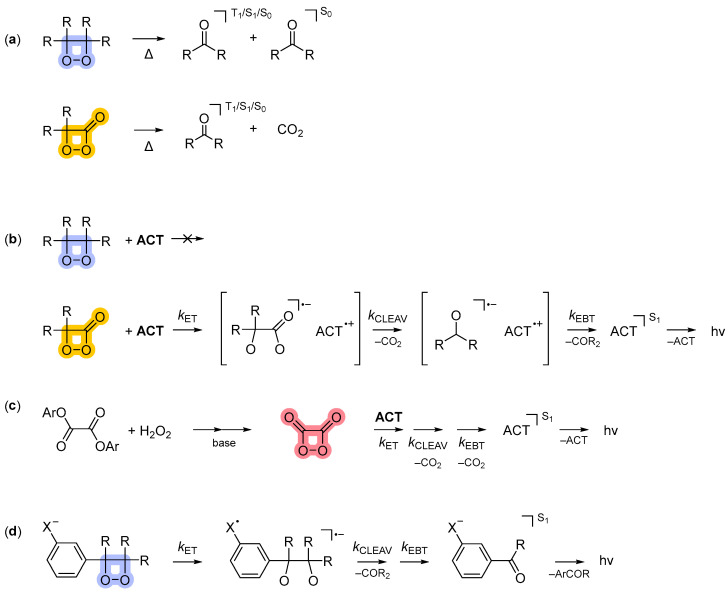
Reaction archetypes in organic chemiluminescence. (**a**) Thermal unimolecular decomposition of 1,2-dioxetanes and 1,2-dioxetanones (intramolecular, inefficient). (**b**) Activated/catalyzed peroxide decomposition of 1,2-dioxetanones (intermolecular, inefficient). (**c**) Peroxyoxalate reaction, formation of 1,2-dioxetanedione as the HEI (intermolecular, efficient). (**d**) Decomposition of electron-rich aryl-substituted 1,2-dioxetanes (intramolecular, efficient). The different types of peroxides are indicated by different colors; 1,2-dioxetane (blue); 1,2-dioxetanone (orange); 1,2-dioxetanone (red).

The unimolecular decomposition of 1,2-dioxetanes and 1,2-dioxetanones leads to the preferential formation of triplet-excited carbonyl compounds (*^3^Φ* up to 50%, *^1^Φ* << 1%, Scheme 3a) [1,5,7]. The direct chemiluminescence of such carbonylic compounds is very inefficient, even in the absence of oxygen in solution, due, in part, to their very low phosphorescence quantum yields. The emission intensity can be enhanced via indirect chemiluminescence using, for example, 9,10-dibromoanthracene (DBA) as an energy acceptor, although this depends on the efficiency of energy transfer (*Φ*^ET^) between the triplet-excited carbonyl compound and the fluorescent dye [1,5,7,16]. 1,2-dioxetanones, but not 1,2-dioxetanes, undergo intermolecular activated chemiluminescence in the presence of an ACT, which involves charge/electron transfer chemistry (Scheme 3b) [17,18,19]. The HEI of the peroxyoxalate reaction, which is 1,2-dioxetandione (**3**), has been shown to react with the ACT in the same way (Scheme 3c) [3,11,15,20,21]. The 1,2-dioxetanes substituted with electron-rich aryl groups undergo intramolecular activated chemiluminescence, leading to the highly efficient formation of singletly excited carbonyl compounds (Scheme 3d) [22,23,24,25,26,27].

### 2.1. Unimolecular Decomposition of 1,2-Dioxetane, 1,2-Dioxetanone, and 1,2-Dioxetanedione

**Scheme 4:**
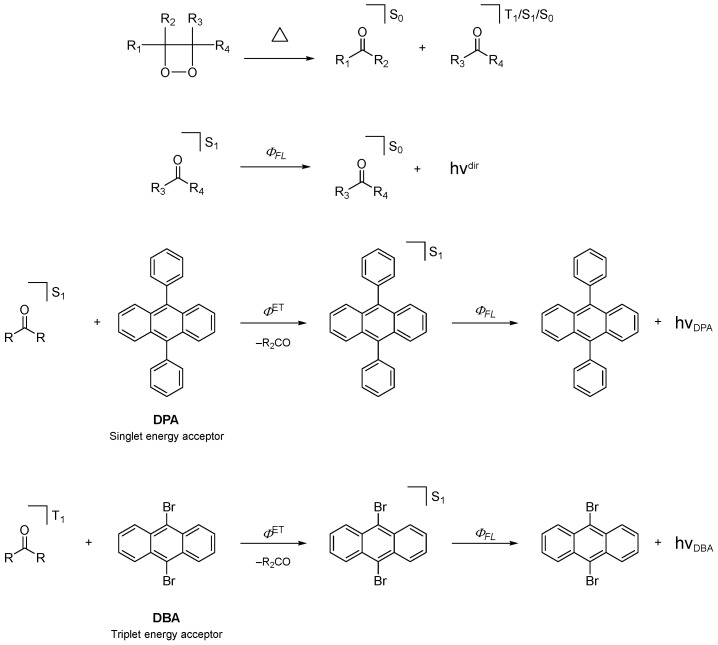
Unimolecular decomposition of 1,2-dioxetanes. Isothermal decomposition kinetics are followed by emission intensity decay and determination of singlet- and triplet-quantum yields using energy transfer to 9,10-diphenylanthracene (DPA) and 9,10-dibromoanthracene (DBA), respectively. *Φ*_Fl_: fluorescence-emission quantum yield; *Φ*_Fl_^DPA^: DPA fluorescence-emission quantum yield; *Φ*_Fl_^DBA^: DBA fluorescence-emission quantum yield; *Φ*_S-S_^ET^: singlet-singlet energy transfer quantum yield; *Φ*_S-T_^ET^: singlet-triplet energy transfer quantum yield; *hν*^dir^: direct CL emission; *hν*^DPA^: DPA sensitized CL emission; *hν*^DBA^: DBA sensitized CL emission.

During the study of chemiluminescent and bioluminescent reactions, several strained cyclic peroxides have been postulated as HEIs. The synthesis and isolation of these compounds are difficult because they are thermolabile and often explosive. Nevertheless, after the first synthesis of a 1,2-dioxetane derivative by Kopecky and Mumford in the late 1960s, several hundreds of derivatives of this class of cyclic peroxides have been synthesized, and the connection between their decomposition mechanism and chemiexcitation efficiency has been extensively studied [2,7,28]. The activation parameters of many 1,2-dioxetanes have been determined by analysis of isothermal kinetics (Scheme 4) [2]. The activation enthalpies for different derivatives span a wide range of about 20 to 35 kcal mol^–1^ (84 to 147 kJ mol^–1^). Activation entropies showed to be close to 0 e.u. [entropy unit (non-SI) = 4.184 J K^–1^ mol^–1^], as expected for a unimolecular process. Reports on negative activation entropy were rationalized considering the presence of impurities (mainly transition metal) able to catalyze peroxide decomposition in solution [5,7,19]. Generally, the presence of bulky substituents stabilizes 1,2-dioxetanes as they hinder the dihedral rotation towards the O–C–C–O angle [7,28,29]. The *^1^Φ* and ^3^*Φ* of 1,2-dioxetanes were determined by using energy transfer to 9,10-diphenylanthracene (DPA) and 9,10-dibromoanthracene (DBA), respectively (Scheme 4). Triplet quantum yields of up to 50% were observed for the decomposition of certain relatively stable derivatives, such as the 3,3,4,4-tetramethyl-1,2-dioxetane (TMD), while singlet quantum yields are generally much lower than 0.1%. Therefore, the thermal decomposition of 1,2-dioxetanes cannot be utilized as a model for efficient BL and CL transformations [2,7,28].

**Scheme 5:**
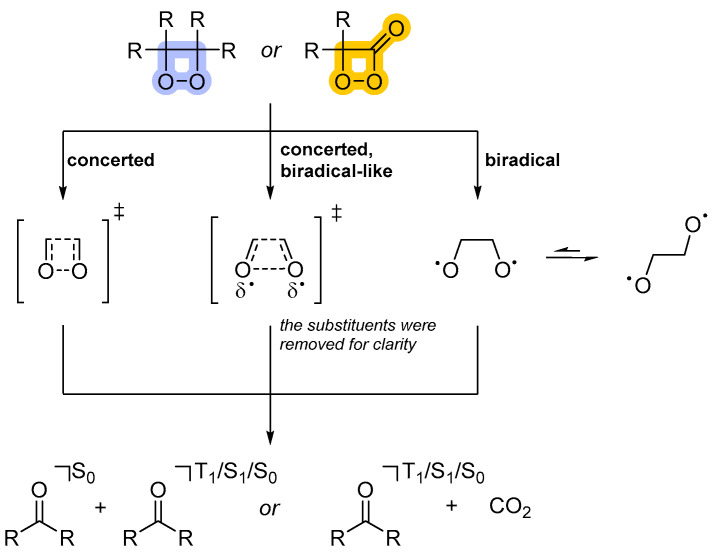
Mechanistic models in thermal (uncatalyzed, unimolecular) 1,2-dioxetane and 1,2-dioxetanone decomposition. The different types of peroxides are indicated by different colors; 1,2-dioxetane (blue); 1,2-dioxetanone (orange).

Different decomposition mechanisms can be formulated for the chemiluminescent cyclic peroxides. Richardson and coworkers proposed that the decomposition of phenyl-substituted 1,2-dioxetanes involves a biradical intermediate as a way to rationalize the observed activation parameters (Scheme 5) [30,31,32]. However, the fact that electronically excited carbonyl compounds are formed in 1,2-dioxetane decomposition can be easily rationalized by the occurrence of a symmetry-forbidden retro-[2+2]-cycloaddition, which should lead to the formation of one of the products in the electronically excited state on the basis of the Woodward Hoffmann rules [33]. In order to explain activation parameters as well as chemiexcitation quantum yields, the “merged” mechanism [34] has been proposed, which is a concerted process but with a biradical nature, as O–O bond cleavage is more advanced than C–C bond cleavage (Scheme 5). The model has been successfully applied to justify activation parameters and quantum yields in the complete series of methyl-substituted 1,2-dioxetanes, including the unsubstituted derivative [35].

Despite the low chemiluminescence efficiency of the thermal decomposition of 1,2-dioxetanes and 1,2-dioxetanones, the fact that these reactions lead to electronically excited products is intriguing. Simple models of chemiexcitation consider that both energetic and geometric requirements must be met for it to occur. In simple terms, (i) the potential energy surface (PES) for the decomposition of the peroxide in the ground state must cross a surface describing the dynamics of the carbonyl product in the excited state, and (ii) this crossing must be kinetically favored over the formation of ground state products, a condition that can be observed if the latter process is so exergonic that chemiexcitation occurs in the inverted region of the Marcus curve. Many theoretical studies have been performed on unimolecular 1,2-dioxetane decomposition, most notably using a series of methyl-substituted derivatives and the unsubstituted 1,2-dioxetane itself. High-level multireference computations revealed that 1,2-dioxetane and its methyl-substituted derivatives should decompose by a concerted biradical-like mechanism [36,37], confirming the hypothesis proposed by the analysis of the experimental data [10,35]. As recently reviewed, these studies also point to the importance of the superposition of relatively flat regions of the PESs for the singlet- and triplet-excited carbonyl products, namely entropic trapping, for efficient triplet chemiexcitation [1]. Most recent theoretical studies describe in detail the conical intersection topologies required to explain the chemiexcitation yields obtained in the series of methyl-substituted 1,2-dioxetanes, where singlet as well as triplet excitation yields increase with the degree of substitution. Dynamic effects appear to be more important for the observation of the different quantum yields than the static features of the potential energy surfaces [38,39].

Contrarily to 1,2-dioxetanes, only a few 1,2-dioxetanones have been synthesized, supposedly due to their still higher instability as compared to 1,2-dioxetanes, caused by the presence of the sp^2^ carbonyl carbon in the four-membered ring [7,40,41]. The unimolecular decomposition of 1,2-dioxetanones, also much less studied, appears to occur similarly to that of 1,2-dioxetanes; however, 1,2-dioxetanones are much less stable and apparently show lower triplet quantum yields upon thermal decomposition [5,7,28,34]. Interestingly, an experimental and theoretical study on the thermal decomposition of spiro-adamantyl-1,2-dioxetanone, the most stable derivative synthesized, indicated that uncatalyzed decomposition occurs with the involvement of different activation energies for ground and excited state formation, confirming results obtained before for 3,3-dimethyl-1,2-dioxetanone [42,43,44,45]. Unsubstituted 1,2-dioxetanone, which could not yet be synthesized, appears to decompose in a stepwise biradical mechanism in the absence of electron transfer catalysis, as demonstrated by multireference computation with the existence of two S_0_/S_1_ conical intersections [46].

No detailed experimental studies on the thermal decomposition of 1,2-dioxetanedione have been conducted, as this peroxide appears only as a highly unstable transient intermediate in peroxyoxalate chemiluminescence [15]. Kinetic studies on its catalyzed decomposition in specific experimental conditions, where this high-energy intermediate can be accumulated in situ, have led to the determination of the activation enthalpy for its unimolecular decomposition (Δ^‡^*H* = 11.2 kcal mol^−1^ = 46.8 kJ mol^−1^), which appears to be too low for its isolation [20]. Theoretical CASPT2 calculations indicate that the decomposition of 1,2-dioxetanedione occurs in a concerted but not simultaneous fashion, contrary to the case of unimolecular 1,2-dioxetanone decompositions for which biradical reaction pathways have been calculated. Additionally, these calculations indicate that the unimolecular decomposition of this peroxide should not lead to efficient excited-state formation, also contrary to the case of 1,2-dioxetanes and 1,2-dioxetanones; however, they are in agreement with the lack of efficient direct emission observed from the peroxyoxalate reaction [47].

### 2.2. Intermolecularly Catalyzed 1,2-Dioxetanone and 1,2-Dioxetanedione Decomposition (Intermolecular CIEEL)

**Scheme 6:**
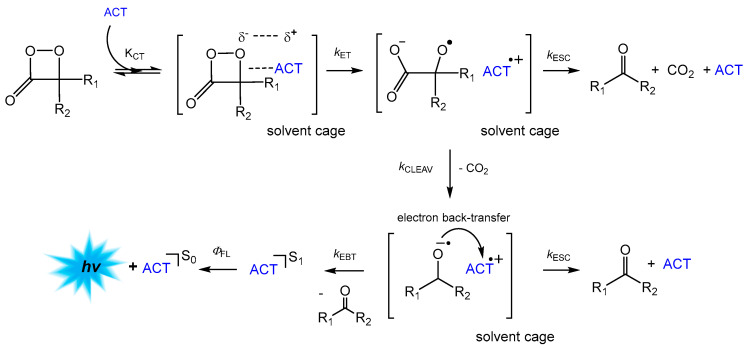
Chemically Initiated Electron Exchange Luminescence (CIEEL) mechanism in the catalyzed decomposition of 1,2-dioxetanone in the presence of an adequate activator (ACT) with low oxidation potential and high fluorescence quantum yields.

During the initial studies on the chemiluminescence characteristics of 1,2-dioxetanone derivatives such as 3,3-dimethyl-1,2-dioxetanone and 3-*tert*-butyl-1,2-dioxetanone, the research groups of W. Adam and G. B. Schuster observed the active role played by the energy acceptors added in the kinetic experiments [44,48,49,50]. Energy acceptors such as DPA and rubrene (initially added to receive the electronic excitation energy of the excited carbonyl compounds formed in the peroxide decomposition) were shown to actively participate in the cyclic peroxide decomposition, and it was verified that the decomposition rate was increased in the presence of these energy acceptors. Additionally, it was demonstrated that the presence of energy acceptors with low oxidation potentials and high fluorescence quantum yields led to an increase in the chemiluminescence quantum yields and that the decomposition rate constants correlated with the oxidation potential of the energy acceptors [17,19]. These experimental findings guided the formulation of the Chemically Initiated Electron Exchange Luminescence (CIEEL) mechanism, where the decomposition of the cyclic peroxide is initiated by an electron transfer from the dye—now called the activator (ACT)—to the peroxide, cleaving the weak O–O bond (*k*_ET_, Scheme 6) [17,19]. The pair of the activator radical cation and the former peroxide radical anion, with the peroxidic bond already cleaved, within the solvent cage can undergo cleavage of the central former ring C–C bond, resulting in a new radical ion pair composed of a carbonyl anion radical and the ACT radical cation (*k*_cleav_). Electron back-transfer (*k*_EBT_) between these radical ions, still within the solvent cage, liberates a neutral carbonyl fragment and can lead to the formation of the ACT in its excited singlet state if the whole process is fast enough to avoid intersystem crossing of the radical ion pair to the triplet state (Scheme 6) [1,5,16,17]. Fluorescence emission from the S_1_ state of the ACT completes the chemiluminescence process, where the ACT acts as an electron transfer catalyst and an efficient emitter of the chemical excitation energy.

It has been suggested that charge-transfer processes, instead of complete electron transfer, might occur in CIEEL; however, no direct experimental evidence of this possibility has been given, and the requirements for either mechanistic alternative are similar; moreover, the term *charge transfer* encompasses *electron transfer* [18]. The CIEEL mechanism has been subsequently utilized to rationalize electronically excited state formation in a wide variety of BL and CL systems, specifically in firefly BL [51]. Additionally, it has been shown experimentally that this chemiexcitation mechanism should operate in the highly efficient peroxyoxalate reaction [3,20,21]. On the other hand, more than thirty years after its original formulation, it has been shown that the standard CIEEL system-catalyzed 1,2-dioxetanone decomposition is occurring with much lower chemiexcitation quantum yields than initially measured, with a difference of around three orders of magnitude [52]. The low quantum yield determined for 3,3-dimethyl-1,2-dioxetanone has also been confirmed for the catalyzed decomposition of the recently synthesized, relatively more stable 1,2-dioxetanone derivatives, namely *spiro*-adamantyl-1,2-dioxetanone and *spiro*-cyclopentyl-1,2-dioxetanones. These low quantum yields could be rationalized by steric hindrance in the initial charge-transfer complex formation necessary for the occurrence of the CIEEL sequence [53,54]. Additionally, it has been shown that the low efficiency of the processes is not due to the cage-escape of radical ion pairs by experimental studies of the solvent viscosity effect on the singlet quantum yield using different solvent mixtures and intermolecular CIEEL systems [55,56,57,58]. A detailed study of the solvent polarity influence on the chemiexcitation efficiency of intermolecular CIEEL systems in different binary polar aprotic and nonpolar solvent systems has shown that an optimum polarity for efficient chemiexcitation exists; however, the polarity influence on the quantum yields is relatively low, indicating low charge separation in the chemiexcitation sequence and favoring the occurrence of a charge transfer, not a complete electron transfer in this mechanism [59]. Contrarily to the inefficient CIEEL systems discussed above, the peroxyoxalate reaction constitutes an intermolecular electron-transfer catalyzed system with verified high quantum yields that can reach close to 100% in optimal conditions [3,4,11,58,59,60].

Theoretical calculations on the catalyzed decomposition of 1,2-dioxetanones have been performed and indicate the occurrence of a partial charge transfer between the peroxide and the activator during chemiexcitation [61]. Additionally, the low singlet-excitation efficiency in catalyzed 1,2-dioxetanone decomposition has been explained based on SA-CASSCF/CASPT2 calculation by efficient competition of the ground-state decomposition channel with chemiexcitation and the formation of non-emissive triplet species [54].

Theoretical studies on the rubrene-catalyzed decomposition of 1,2-dioxetanedione indicated the occurrence of a stepwise electron-transfer and electron back-transfer mechanism including various S_0_/S_1_ conical intersections in the different electron-transfer states, which could lead to chemiexcitation of the activator [39,62]. This approach allowed it to estimate a singlet excitation quantum yield of around 50%, in reasonable agreement with experimental data for different conditions [3,4,11,58,59,60].

### 2.3. Intramolecularly Catalyzed 1,2-Dioxetane Decomposition (Intramolecular CIEEL)

Although 1,2-dioxetanes do not show catalyzed decomposition in the presence of external activators, if an electron-donating unit is covalently linked to the peroxidic ring, the derivative undergoes induced decomposition, which is apparently initiated by an intramolecular electron- or charge-transfer process. The first example of the induced decomposition of 1,2-dioxetanes was reported by the P. Schaap research group when they found out that 1,2-dioxetane derivatives containing phenolic substituents show ultrafast decomposition upon deprotonation, resulting in the efficient formation of singlet-excited carbonyl products [22]. Phenoxy-substituted 1,2-dioxetane derivatives, containing adequate protective groups of the phenolate moiety and generally a *spiro*-adamantyl group for stabilization, have subsequently been developed as CL probes for detecting various analytes [22,63,64], most prominently in CL detection systems for different immunoassays [23,65,66,67,68].

Many 1,2-dioxetane derivatives containing protected electron-rich moieties have been synthesized and their decomposition mechanisms and chemiexcitation efficiency have been studied [26]. The general design of these 1,2-dioxetane derivatives, whose decomposition can be induced by adequate triggering agents, leading to efficient singlet-excited state formation and high CL emission, consists of (i) the 1,2-dioxetane ring as a high-energy moiety; (ii) a stabilizing unit with high steric impact, conferring relatively high thermal stability to the peroxide; and (iii) the protected trigger moiety, which can be transformed into the electron-donating unit upon chemical or enzymatic deprotection [23]. Generally, the 1,2-dioxetane moiety consists of a tri-alky- and/or -aryl substituted 1,2-dioxetane, still containing at least one electron-donating group linked to the peroxide ring (such as an alkoxy-group) to allow convenient synthesis of the cyclic peroxide by photooxygenation [23,26,69,70]. Alternatively, 1,2-dioxetanes not containing heteroatom substituents at the peroxidic ring can also be used for the induced dioxetane decomposition, where the cyclic peroxides have to be synthesized by the conventional Kopecky method [24,25]. The *spiro*-adamantyl-substituent has been initially utilized as the stabilizing group, most notably in the 3-(2′-spiro-adamantyl)-4-methoxy-4-(3″-phosphoryloxy)-phenyl-1,2-dioxetane (*m*-AMPPD), used as the detecting device in a wide variety of commercially available immunoassays. Alternatively, substitution by two isopropyls on the peroxidic ring has significantly increased thermal stability, and several 3,3-diisopropyl substituted 1,2-dioxetane derivatives subject to induced decomposition have been synthesized and studied [71,72]. Other sterically demanding groups have been used for the stabilization of the peroxidic ring in triggerable 1,2-dioxetanes, including the fenchyl group [73] as well as the utilization of bicyclic derivatives [74,75,76,77]. The most utilized phenolate protecting group for mechanistic studies has been the trialkylsilyl group, which can be detached specifically from the fluoride ion [26]. In contrast, several protective groups have been utilized for specific deprotection by enzymes, such as alkaline phosphatase and many other enzymes [78]. More recently, protective groups have been utilized in synthesizing 1,2-dioxetane derivatives that can be specifically deprotected by chemical analytes or enzymes [79]. The induced decomposition of phenoxy-substituted and equivalent 1,2-dioxetanes has been shown to occur with extremely high singlet quantum yields, being able to reach up to 100% [4,24,25].

**Scheme 7:**
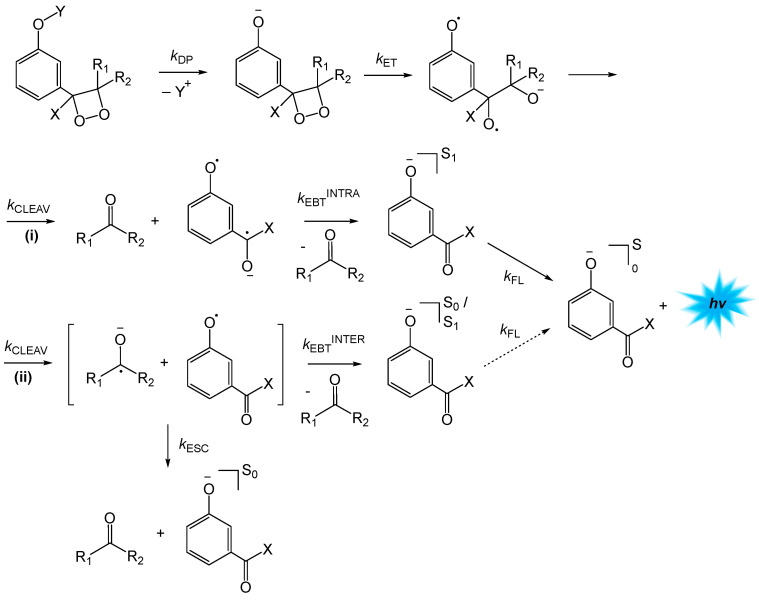
Mechanism of the induced decomposition of phenoxy-substituted 1,2-dioxetanes. R_1_, R_2_: alkyl; R_1_-R_2_: *spiro*-adamantyl; X: -OCH_3_, H; Y: protection group; k_DP_: deprotection rate constant; *k*_ET_: electron-transfer rate constant; *k*_CLEAV_: C-C bond cleavage rate constant; *k*_EBT_^INTRA^: intramolecular electron back-transfer rate constant; *k*_EBT_^INTER^: intermolecular electron back-transfer rate constant; *k*_ESC_: radical pair solvent-cage escape rate constant; *k*_Fl_: fluorescence emission rate constant.

The mechanism of the induced decomposition of phenoxy-substituted 1,2-dioxetanes can be formulated in analogy to the intermolecular CIEEL, with the difference that the initial electron- or charge-transfer step occurs in an intramolecular manner, not involving an external activator (Scheme 7) [5]. The electron transfer from the phenolate oxygen, formed by deprotection of the silyl-protected phenolic moiety (*k*_DP_), to the O-O bond is accompanied by O–O bond cleavage, leading to the formation of a diradical anion (*k*_ET_), with the unpaired electron and the negative charge being located at any of the oxygen atoms of the former peroxide. Cleavage of the central C–C bond (*k*_cleav_) can occur in two distinct ways: (i) leaving a neutral carbonyl compound and a formal biradical anion, or (ii) giving rise to a carbonyl radical anion and a phenolate radical (Scheme 7). The biradical anion formed in the pathway (i) undergoes a formal intramolecular electron transfer (*k*_EBT_^INTRA^), giving rise to the singlet-excited carbonyl product, which may be the reason for the high chemiexcitation efficiency of this process. In pathway (ii), the radical pair has to undergo an intermolecular electron transfer for product formation (*k*_EBT_^INTER^), which could also lead to a singlet-excited product, however with lower efficiency and mainly ground-state formation; escape of the radical pair formed from the solvent cage will always lead to ground-state product formation. Fluorescence emission from the S_1_ state of the product completes the CL sequence (Scheme 7).

The occurrence of an electron transfer in the induced 1,2-dioxetane decomposition has been demonstrated experimentally in a Hammett study on the decomposition of acridinium-substituted 1,2-dioxetanes containing additionally a phenyl group bearing substituents with different electronic properties. The correlation of the decomposition rate constants with the Hammett substituent parameters (σ) yielded Hammett reaction constants, indicating the formation of a significant negative charge in the rate-limiting step of the transformation, meaning the occurrence of an electron transfer process [80,81]. Nonetheless, it has been suggested that charge-transfer interactions could occur instead of full electron-transfer steps, mainly based on the results of theoretical calculations [18,61,82]; however, it appears challenging to find an experimental approach to differentiate between these.

The high singlet quantum yields clearly favor the occurrence of the entirely intramolecular pathway, where the efficiency cannot be decreased by radical pair solvent cage escape. Contrarily, the experimental observation that singlet quantum yields in induced 1,2-dioxetane decomposition increased with the solvent viscosity appeared to indicate the occurrence of an intermolecular electron back-transfer [83,84,85]. However, more recent studies on the induced 1,2-dioxetane decomposition (intramolecular CIEEL) and the peroxyoxalate system (intermolecular CIEEL) indicated that the observed solvent-cage effect on the quantum yields of induced 1,2-dioxetane decomposition could be rationalized with the necessity of a specific conformation of the diradical pair for efficient chemiexcitation in the electron back-transfer step [86]. This conclusion of an entirely intramolecular transformation in the induced decomposition of phenoxy-substituted 1,2-dioxetanes has been further sustained by studies on the solvent viscosity effects on several inter- and intramolecular CIEEL systems, whose results could be justified by an intramolecular electron back-transfer step in the induced 1,2-dioxetane decomposition [55,56,57]. Additionally, a most recent study of the solvent polarity effect on the chemiexcitation efficiency of inter- and intramolecular CIEEL chemiexcitation systems, including catalyzed 1,2-dioxetanone decomposition, and induced 1,2-dioxetane decomposition indicated that in the latter transformation, the electron back-transfer should occur in an intramolecular fashion, whereas in the former, this process should be intermolecular [59].

Another interesting point in induced 1,2-dioxetane decomposition is that to obtain highly efficient chemiexcitation, the phenoxy-substituent should bear the phenolic oxygen atom in *meta* position with respect to the connection to the peroxidic ring. This so-called *meta* effect or odd-even selection rule (when applied to naphthyl or other substituents) states that when the substituent is in direct conjugation with the peroxidic ring (*para* position in phenyl or even in naphthyl), the quantum yields are much lower. However, induced decomposition is generally faster with a substituent not directly conjugated with the peroxidic ring (*meta* position in phenyl or odd position in naphthyl) [85,87]. A study on the decomposition kinetics and the quantum yields of the regioisomeric 1,2-dioxetanes m-AMPPD and its *para* regioisomer, *p*-AMPD [4′-(4-hydroxyphenyl)-4′-methoxyadamantanespiro-1,2-dioxetane], revealed that *m*-AMPPD exhibits a steady glow of light emission for several minutes, whereas p-AMPPD releases a flash of light with a drastic difference in the emission quantum yields, with the *m*-isomer being 10^4^ times more efficient [85]. The reason for this drastic dependence of the CL properties on the substitution pattern is unclear yet, although several theoretical investigations have been performed on the subject [1]. One possible explanation could be that in the *para* isomer, the π* orbital of the carbonyl interacts with the HOMO of the phenoxide group due to orbital symmetry; therefore, the *para* isomer collapses directly from the electron-transfer biradical anion intermediate to the ground state of the donor [1]. Additionally, the differences between the CL properties of the *meta* and *para* isomers of 1,2-dioxetanone derivatives containing phenoxy anion substituents have been rationalized using similar orbital symmetry rule arguments [61,82].

### 2.4. Chemiluminescence Transformations Applied to Analytical Assays

As already mentioned, many CL transformations are applied in numerous analytical and bioanalytical transformations. From the basic general chemiexcitation mechanisms outlined before, the most promising one for analytical and imaging applications is the induced decomposition of properly substituted 1,2-dioxetanes, which can suffer an intramolecular CIEEL mechanism, as these transformations commonly occur with extremely high singlet-excitation quantum yields [79,88,89,90]. The unimolecular decomposition of 1,2-dioxetanes is not a proper system for analytical applications, as this process leads mainly to the formation of non-emissive triplet excited products [1,5,7,35]. The catalyzed decomposition of 1,2-dioxetanones and related cyclic peroxides occurs with the preferential formation of emissive singlet excited products; however, the singlet quantum yields in this intermolecular CIEEL system show generally low singlet-excitation quantum yields, not suitable for analytical applications [4,17,19,52,53]. Contrarily, the peroxyoxalate reaction, where 1,2-dioxetanedione appears to be formed as a high-energy intermediate [15], proceeds with extremely high singlet-excitation quantum yields and is known to occur by the intermolecular CIEEL mechanism [3,4,20]. Therefore, this transformation is highly adequate for analytical applications.

Apart from these “classical” CL systems extensively studied in the past to establish the general chemiexcitation mechanisms, several complex CL transformations have found analytical applications and should occur by chemiexcitation mechanisms similar to the intramolecular CIEEL [1,4,5]. In many of these transformations, the chemiexcitation mechanism operating is not exactly known, but they usually show emission quantum yields in the 10% range [1,5]. Examples of this kind of transformation are the long-known oxidations of luminol [91,92,93,94,95], lophine [96,97,98,99], and lucigenin [100,101,102,103], as well as the similar reaction of acridinium esters [104,105,106,107].

The CL emission spectrum obtained in the different transformations is not related to the chemiexcitation mechanism but simply to the fluorescence spectrum of the reaction product formed in its excited singlet state or the excitation energy acceptor present in the system that receives the excitation energy from the initially formed excited product. In the peroxyoxalate reaction, the CL emission wavelength can be chosen almost at will by the choice of the emission spectrum of the activator used, which is chemiexcited by interaction with the high-energy intermediate formed in the reaction sequence [108,109,110,111,112].

In the induced 1,2-dioxetane decomposition, the emission wavelength of the dioxetane-decomposition product has been chosen by the proper substitution of the 1,2-dioxetane derivative utilized [113,114,115,116]. Additionally, the emission spectra can be selected by the addition of appropriate energy acceptors that receive the excitation energy from the 1,2-dioxetane decomposition product, and the CL emission spectrum will correspond to the fluorescence spectrum of the energy acceptor [113,114,115].

Finally, it must be mentioned that many BL transformations have been utilized as detection systems in bioanalytical applications and bioimaging [116,117,118,119]. These BL systems include the well-studied firefly BL, where an intramolecular CIEEL-like chemiexcitation mechanism appears to operate, and the coelenterazine BL, where different mechanisms are postulated [1,51,120,121,122]. However, these BL transformations and their applications are clearly out of the scope of this contribution and are therefore only mentioned here.

## 3. Applications of CL Transformations in Bioassays

Reaction-based chemiluminescent probes have received enormous attention over the past decade due to their use in a range of high-impact applications, including the detection of target molecules in living cells with higher emission intensities and increased selectivity. Compared to other luminescence phenomena, the most important advantage of chemiluminescence assays is the low background noise produced by eliminating an external light source, providing, therefore, more opportunities for in vivoimaging. For this reason, chemiluminescence for biological use was registered as one of the IUPAC’s Top Ten Emerging Technologies in Chemistry in 2021 [123]. Herein, we will mention some recent representative examples of using chemiluminescence in bioassays.

**Scheme 8:**
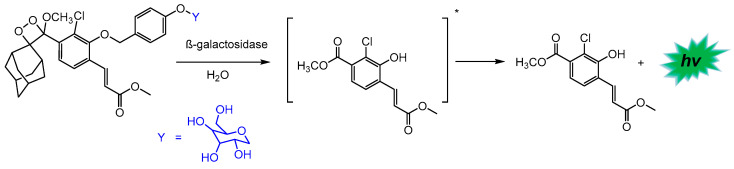
Chemiluminescence probe for the detection of β-galactosidase. Adapted with permission from Ref. [126]. Copyright 2017, American Chemical Society. The * indicates that the compound is formed in its singlet-excited state.

Lippert and co-workers were among the first to report studies demonstrating the in vivo compatibility of various chemiluminescent systems [124,125]. Since then, there has been an increase in research activity in this field in order to preserve the chemiluminescence properties of these systems under biologically relevant conditions. One example of this is the work of Shabat’s group, which demonstrated that the incorporation of a substituent to the benzoate moiety of Schaap’s 1,2-dioxetane led to a significant increase in chemiluminescence emission intensity under physiological conditions [126]. This allowed it to provide, for the first time, a probe for cell imaging with the direct chemiluminescence emission mode based on the endogenous activity of β-galactosidase other than firefly luciferin (Scheme 8).

**Scheme 9:**
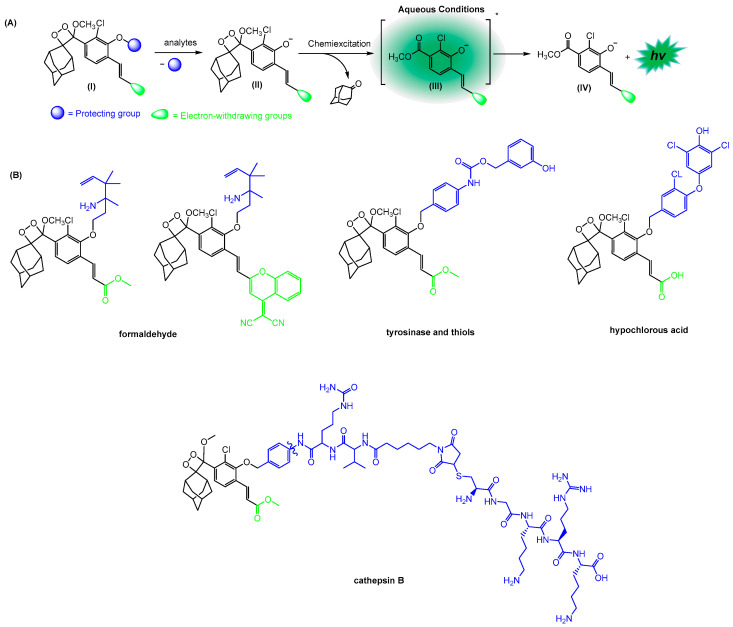
(**A**) The general chemiluminescence activation pathway of probes allows for detecting different analytes. (**B**) Structures of the chemiluminescence probes and names of detected analytes Adapted with permission from reference [130]. Copyright 2021, Chinese Chemical Society. The * indicates that the compound is formed in its singlet-excited state.

Based on this strategy of introducing significant structural modification in 1,2-dioxetanes, they also developed several chemiluminescence probes for detecting and imaging different analytes such as cathepsin B [127], tyrosinase and thiols [128], formaldehyde [129], and hypochlorous acid [130], among others (Scheme 9) [131].

The phenol moiety of these probes is masked by an analyte-responsive protecting group (Structure I). Furthermore, the deprotection generates the unstable phenolate-dioxetane (II), which decomposes via the CIEEL mechanism to form the excited intermediate benzoate ester (III). Finally, the decay to its ground state (IV) is accompanied by the emission of light that is correlated with the analyte activity (Scheme 9).

**Scheme 10:**
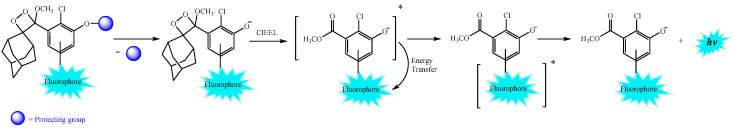
Proposed mechanism of the chemiluminescence activation pathway of dioxetane−fluorophore conjugate. Adapted with permission from ref [113]. Copyright 2022, American Chemical Society. Ref [114]. Copyright 2020, American Chemical Society and ref. [132], John Wiley & Sons. The * indicates that the compound is formed in its singlet-excited state.

Recently, Lippert’s group has also shown the versatility of linking several fluorophores with the acrylamide 1,2-dioxetane chemiluminescent scaffold for radiometric imaging of pH and oxygen concentration in live animals [114,132] as well as for dark photodynamic therapy [113]. In all these cases, the initial removal of the protection group by the analyte of interest occurs to start the CIEEL mechanism. Then, the excited benzoate formed in the chemiexcitation step transfers its energy to the fluorophore, resulting in excitation of the latter and then light emission at its specific emission wavelength (Scheme 10).

**Scheme 11:**
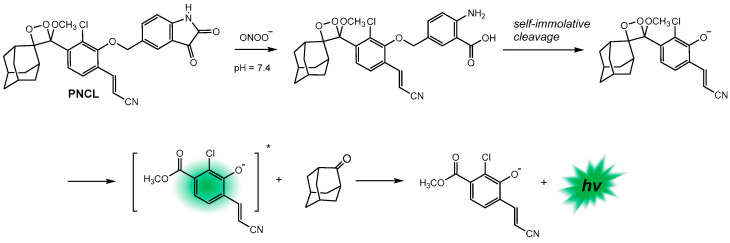
The chemiluminescence reagent PNCL for ONOO^–^ detection. Adapted with permission from ref. [134]. Copyright 2018, The Royal Society of Chemistry. The * indicates that the compound is formed in its singlet-excited state.

The same research group also synthesized a hydrazinyl naphthalimide fluorescent probe to directly visualize endogenous carbonyl metabolites [133] and developed an isatin-based chemiluminescent probe for peroxynitrite detection (PNCL) in aqueous solution and living cells [134]. In this last case, the PNCL probe reacts with peroxynitrite (ONOO^–^) through an oxidative decarbonylation reaction with high analytical sensitivity and specificity. This is followed by a rapid and spontaneous self-immolation via 1,2-dioxetane cleavage with efficient chemiexcitation according to the CIEEL mechanism, resulting in high-intensity light emission (Scheme 11).

**Scheme 12:**
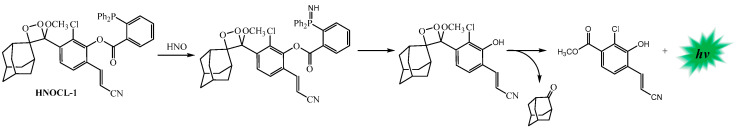
Reactions for the chemiluminescence emission of HNOCL-1. Adapted with permission from ref. [135]. Copyright 2019, John Wiley & Sons.

A short time later, the first chemiluminescent probe for azanone (HNO) (HNOCL-1) in living systems based on a reaction with a phosphine group to form a self-cleavable azaylide intermediate was synthesized (Scheme 12) [135].

**Scheme 13:**
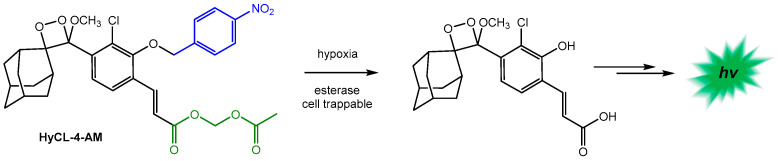
The structure and sensing reaction for the HyCL-4-AM probe. Adopted with permission from ref. [136]. Copyright 2019, American Chemical Society.

Another significant and recent contribution was the design of probes as chemiluminescent indicators of hypoxia in cellular and animal models [136]. One of these probes, containing a nitroaromatic sensing moiety and an acetoxymethyl ester moiety (HyCL-4-AM), exhibited high chemiluminescence emission, demonstrating its ability to measure and image hypoxia in tumor xenograft models (Scheme 13).

**Scheme 14:**
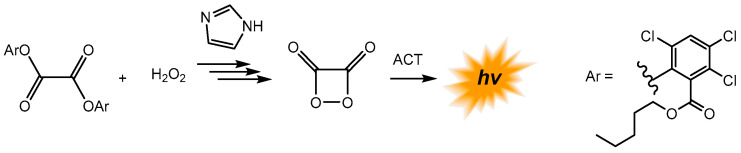
The peroxyoxalate reaction for H_2_O_2_ quantification in the exhaled breath condensate of asthma patients. Adopted with permission from ref. [137]. Copyright 2020, American Chemical Society.

Furthermore, the peroxyoxalate system was recently used by Lippert and coworkers to measure hydrogen peroxide (H_2_O_2_) in the exhaled breath condensate of asthma patients [137]. This study uses bis(2-carbopentyloxy-3,5,6-trichlorophenyl) oxalate (CPPO) as an oxalic ester, imidazole as a base catalyst, and rubrene as a fluorophore that is chemically excited by the high-energy intermediate, 1,2-dioxetanedione, formed from the oxalic ester and hydrogen peroxide (Scheme 14).

**Scheme 15:**
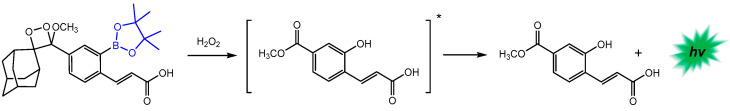
The reaction of the arylboronate-substituted 1,2-dioxetane probe for chemiluminescent H_2_O_2_ quantification. Adopted with permission from ref. [138]. Copyright 2020, Elsevier. The * indicates that the compound is formed in its singlet-excited state.

On the other hand, Calabria and co-workers developed a chemiluminescent bioassay based on an adamantylidene-1,2-dioxetane probe containing an arylboronate moiety for the quantification of intracellular H_2_O_2_ in human living cells [138]. This probe is converted to the correspondent phenol upon reaction with H_2_O_2_, which emits light through the CIEEL mechanism with high efficiency (Scheme 15).

Despite the exciting progress in chemiluminescence, as mentioned above, critical challenges, such as increasing the limited number of molecular scaffolds used, remain in this field. In order to solve this issue, many researchers will undoubtedly be rewarded with discoveries on this subject in the years to come.

## 4. Conclusions

In this review article, we describe the general characteristics and requirements of chemiluminescence transformations, outline the main types of these transformations, and give some useful definitions in CL research. The main general chemiexcitation mechanisms, which are valid for most if not all CL and BL transformations, are then discussed in detail, including experimental and theoretical results that support these mechanistic proposals. Special emphasis is given to the structural requirements for each mechanism and their singlet and triplet excitation efficiencies. Finally, to illustrate the application potential of CL transformations, we give some recent applications of CL transformations in chemical and biochemical analysis as well as bioimaging.

## Data Availability

Due to the nature of this article as a review, no data supporting reported results are available.

## References

[1] Vacher M., Fdez Galván I., Ding B.W., Schramm S., Berraud-Pache R., Naumov P., Ferré N., Liu Y.J., Navizet I., Roca-Sanjuán D. (2018). Chemi- and Bioluminescence of Cyclic Peroxides. Chem. Rev..

[2] El Seoud O.A., Baader W.J., Bastos E.L., Wang Z. (2016). Encyclopedia of Physical Organic Chemistry, 5 Volume Set.

[3] Stevani C.V., Silva S.M., Baader W.J. (2000). Studies on the Mechanism of the Excitation Step in Peroxyoxalate Chemiluminescence. Eur. J. Org. Chem..

[4] Augusto F.A., De Souza G.A., De Souza S.P., Khalid M., Baader W.J. (2013). Efficiency of Electron Transfer Initiated Chemiluminescence. Photochem. Photobiol..

[5] Baader W.J., Stevani C.V., Bastos E.L., Rappoport Z. (2006). Chemiluminescence of Organic Peroxides. The Chemistry of Peroxides.

[6] Ando Y., Niwa K., Yamada N., Enomoto T., Irie T., Kubota H., Ohmiya Y., Akiyama H. (2008). Firefly Bioluminescence Quantum Yield and Colour Change by PH-Sensitive Green Emission. Nat. Photonics.

[7] Adam W., Cilento G. (1982). Chemical and Biological Generation of Excited States.

[8] Kopecky K.R., Mumford C. (1969). Luminescence in the Thermal Decomposition of 3,3,4-Trimethyl-1,2-Dioxetane. Can. J. Chem..

[9] Adam W., Liu J.-C. (1972). Cyclic Peroxides. XVI. Alpha-Peroxy Lactone. Synthesis and Chemiluminescence. J. Am. Chem. Soc..

[10] Adam W., Baader W.J. (1984). 1,2-Dioxetane: Synthesis, Characterization, Stability, and Chemiluminescence. Angew. Chem. Int. Ed. Engl..

[11] Rauhut M.M. (1969). Chemiluminescence from Concerted Peroxide Decomposition Reactions. Acc. Chem. Res..

[12] Stevani ‪C.V., Campos I.P.D.A., Baader W.J. (1996). Synthesis and Characterisation of an Intermediate in the Peroxyoxalate Chemiluminescence: 4-Chlorophenyl O,O-Hydrogen Monoperoxy-Oxalate. J. Chem. Soc. Perkin Trans..

[13] Stevani ‪C.V., Baader W.J. (1997). Kinetic Studies on the Chemiluminescent Decomposition of an Isolated Intermediate in the Peroxyoxalate Reaction. J. Phys. Org. Chem..

[14] Tonkin S.A., Bos R., Dyson G.A., Lim K.F., Russell R.A., Watson S.P., Hindson C.M., Barnett N.W. (2008). Studies on the Mechanism of the Peroxyoxalate Chemiluminescence Reaction. Part 2. Further Identification of Intermediates Using 2D EXSY13C Nuclear Magnetic Resonance Spectroscopy. Anal. Chim. Acta..

[15] da Silva S.M., Lang A.P., dos Santos A.P.F., Cabello M.C., Ciscato L.F.M.L., Bartoloni F.H., Bastos E.L., Baader W.J. (2021). Cyclic Peroxidic Carbon Dioxide Dimer Fuels Peroxyoxalate Chemiluminescence. J. Org. Chem..

[16] Bastos E.L., Farahani P., Bechara E.J.H., Baader W.J. (2017). Four-Membered Cyclic Peroxides: Carriers of Chemical Energy. J. Phys. Org. Chem..

[17] Schuster G.B. (1979). Chemiluminescence of Organic Peroxides. Conversion of Ground-State Reactants to Excited-State Products by the Chemically Initiated Electron-Exchange Luminescence Mechanism. Acc. Chem. Res..

[18] Wilson T. (1995). Comments on the Mechanisms of Chemi- and Bioluminescence. Photochem. Photobiol..

[19] Schuster G.B., Schmidt S.P. (1982). Chemiluminescence of Organic Compounds. Adv. Phys. Org. Chem..

[20] Ciscato L.F.M.L., Bartoloni F.H., Bastos E.L., Baader W.J. (2009). Direct Kinetic Observation of the Chemiexcitation Step in Peroxyoxalate Chemiluminescence. J. Org. Chem..

[21] Silva S.M., Wagner K., Weiss D., Beckert R., Stevani C.V., Baader W.J. (2002). Studies on the Chemiexcitation Step in Peroxyoxalate Chemiluminescence Using Steroid-Substituted Activators. Luminescence.

[22] Schaap A.P., Gagnon S.D. (1982). Chemiluminescence from a Phenoxide-Substituted 1,2-Dioxetane: A Model for Firefly Bioluminescence. J. Am. Chem. Soc..

[23] Adam W., Reinhardt D., Saha-Möller C.R. (1996). From the Firefly Bioluminescence to the Dioxetane-Based (AMPPD) Chemiluminescence Immunoassay: A Retroanalysis. Analyst.

[24] Nery A.L.P., Röpke S., Catalani L.H., Baader W.J. (1999). Fluoride-Triggered Decomposition of m-Sililoxyphenyl-Substituted Dioxetanes by an Intramolecular Electron Transfer (CIEEL) Mechanism. Tetrahedron Lett..

[25] Nery A.L.P., Weiss D., Catalani L.H., Baader W.J. (2000). Studies on the Intramolecular Electron Transfer Catalyzed Thermolysis of 1,2-Dioxetanes. Tetrahedron.

[26] Matsumoto M. (2004). Advanced Chemistry of Dioxetane-Based Chemiluminescent Substrates Originating from Bioluminescence. J. Photochem. Photobiol. C Photochem. Rev..

[27] Nery A.L.P., Baader W.J. (2001). Quimiluminescência de Peróxidos Orgânicos: Geração de Estados Eletronicamente Excitados Na Decomposição de 1,2-Dioxetanos. Quim. Nova.

[28] Adam W., Trofimov A.V., Rappoport Z. (2006). The Chemistry of Peroxides.

[29] Bastos E.L., Baader W.J. (2007). Theoretical Studies on Thermal Stability of Alkyl-Substituted 1,2-Dioxetanes. Arkivoc.

[30] Richardson W.H., O’Neal H.E. (1972). The Unimolecular Decomposition and Isomerization of Oxygenated Organic Compounds (Other than Aldehydes and Ketones). Compr. Chem. Kinet..

[31] Richardson W.H., Lovett M.B., Price M.E., Anderegg J.H. (1979). Excited-State Energy Distribution Between Dissimilar Carbonyl Molecules Produced from 1, 2-Dioxetanes. J. Am. Chem. Soc..

[32] Richardson W.H., Batinica G., Janota-Perret K., Miller T., Shen D. (1991). Excited State Selectivity in the Thermolysis of a 3,4-Diaryl-3,4-Dimethyl-l,2-Dioxetane. J. Org. Chem..

[33] McCapra F. (1968). An Application of the Theory of Electrocyclic Reactions to Bioluminescence. Chem. Commun..

[34] Adam W. (1980). Thermal Generation of Electronic Excitation with Hyperenergetic Molecules. Pure Appl. Chem..

[35] Adam W., Baader W.J. (1985). Effects of Methylation on the Thermal Stability and Chemiluminescence Properties of 1,2-Dioxetanes. J. Am. Chem. Soc..

[36] de Vico L., Liu Y.J., Krogh J.W., Lindh R. (2007). Chemiluminescence of 1,2-Dioxetane. Reaction Mechanism Uncovered. J. Phys. Chem. A.

[37] Farahani P., Roca-Sanjuán D., Zapata F., Lindh R. (2013). Revisiting the Nonadiabatic Process in 1,2-Dioxetane. J. Chem. Theory Comput..

[38] Fdez. Galván I., Brakestad A., Vacher M. (2022). Role of Conical Intersection Seam Topography in the Chemiexcitation of 1,2-Dioxetanes. Phys. Chem. Chem. Phys..

[39] Yue L., Liu Y.-J. (2022). Conical Intersection in Chemiluminescence of Cyclic Peroxides. J. Phys. Chem. Lett..

[40] Adam W., Blancafort L. (1997). Reaction of α-Peroxy Lactones with C, N, P, and S Nucleophiles: Adduct Formation and Nucleophile Oxidation by Nucleophilic Attack at and Biphilic Insertion into the Peroxide Bond. J. Org. Chem..

[41] Bartoloni F.H., de Oliveira M.A., Augusto F.A., Ciscato L.F.M.L., Bastos E.L., Baader W.J. (2012). Synthesis of Unstable Cyclic Peroxides for Chemiluminescence Studies. J. Braz. Chem. Soc..

[42] Farahani P., Oliveira M.A., Fdez GalvánGalv I., Bc G., Baader W.J. (2017). A Combined Theoretical and Experimental Study on the Mechanism of Spiro-Adamantyl-1,2-Dioxetanone Decomposition. RSC Adv..

[43] Steinmetzer H.C., Yekta A., Turro N.J. (1974). Chemiluminescence of Tetramethyl-1,2-Dioxetane. Measurement of Activation Parameters and Rates of Exceedingly Slow Reactions by a Simple and “Nondestructive” Method. Demonstration of Indistinguishable Activation Energies for Generation of Acetone Singlets and Triplets. J. Am. Chem. Soc..

[44] Schmidt S.P., Schuster G.B. (1978). Kinetics of Unimolecular Dioxetanone Chemiluminescence. Competitive Parallel Reaction Paths. J. Am. Chem. Soc..

[45] Turro N.J., Chow M.F. (1980). Chemiluminescent Thermolysis of α-Peroxylactones. J. Am. Chem. Soc..

[46] Liu F., Liu Y., de Vico L., Lindh R. (2009). Theoretical Study of the Chemiluminescent Decomposition of Dioxetanone. J. Am. Chem. Soc..

[47] Farahani P., Baader W.J. (2017). Unimolecular Decomposition Mechanism of 1,2-Dioxetanedione: Concerted or Biradical? That Is the Question!. J. Phys. Chem. A..

[48] Adam W., Simpson G.A., Yany F. (1974). Mechanism of Direct and Rubrene Enhanced Chemiluminescence during α-Peroxylactone Decarboxylation. J. Phys. Chem..

[49] Adam W., Cueto O. (1979). Fluorescer-Enhanced Chemiluminescence of α-Peroxylactones via Electron Exchange. J. Am. Chem. Soc..

[50] Schmidt S.P., Schuster G.B. (1980). Chemiluminescence of Dimethyldioxetanone. Unimolecular Generation of Excited Singlet and Triplet Acetone. Chemically Initiated Electron-Exchange Luminescence, the Primary Light Generating Reaction. J. Am. Chem. Soc..

[51] Koo J.A., Schmidt S.P., Schuster G.B. (1978). Bioluminescence of the Firefly: Key Steps in the Formation of the Electronically Excited State for Model Systems. Proc. Natl. Acad. Sci. USA.

[52] Almeida De Oliveira M., Bartoloni F.H., Augusto F.A., Ciscato L.F.M.L., Bastos E.L., Baader W.J. (2012). Revision of Singlet Quantum Yields in the Catalyzed Decomposition of Cyclic Peroxides. J. Org. Chem..

[53] Bartoloni F.H., De Oliveira M.A., Ciscato L.F.M.L., Augusto F.A., Bastos E.L., Baader W.J. (2015). Chemiluminescence Efficiency of Catalyzed 1,2-Dioxetanone Decomposition Determined by Steric Effects. J. Org. Chem..

[54] Augusto F.A., Francés-Monerris A., Fdez Galván I., Roca-Sanjuán D., Bastos E.L., Baader W.J., Lindh R. (2017). Mechanism of Activated Chemiluminescence of Cyclic Peroxides: 1,2-Dioxetanes and 1,2-Dioxetanones. Phys. Chem. Chem. Phys..

[55] Khalid M., de Souza S.P., Bartoloni F.H., Augusto F.A., Baader W.J. (2016). Chemiexcitation Efficiency of Intermolecular Electron-Transfer Catalyzed Peroxide Decomposition Shows Low Sensitivity to Solvent-Cavity Effects. Photochem. Photobiol..

[56] Khalid M., Oliveira M.A., Souza S.P., Ciscato L.F.M.L., Bartoloni F.H., Baader W.J. (2015). Efficiency of Intermolecular Chemiluminescence Systems Lacks Significant Solvent Cavity Effect in Binary Toluene/Diphenylmethane Mixtures. J. Photochem. Photobiol. A Chem..

[57] Khalid M., Souza S.P., Ciscato L.F.M.L., Bartoloni F.H., Baader W.J. (2015). Solvent Viscosity Influence on the Chemiexcitation Efficiency of Inter and Intramolecular Chemiluminescence Systems. Photochem. Photobiol. Sci..

[58] Souza S.P., Khalid M., Augusto F.A., Baader W.J. (2016). Peroxyoxalate Chemiluminescence Efficiency in Polar Medium Is Moderately Enhanced by Solvent Viscosity. J. Photochem. Photobiol. A Chem..

[59] Khalid M., Souza S.P., Cabello M.C., Bartoloni F.H., Ciscato L.F.M.L., Bastos E.L., Seoud O.A.A.E., Baader W.J. (2022). Solvent Polarity Influence on Chemiexcitation Efficiency of Inter and Intramolecular Electron-Transfer Catalyzed Chemiluminescence. J. Photochem. Photobiol. A Chem..

[60] Catherallt C.L.R., Palmer T.F. (1989). Determination of Absolute Chemiluminescence Quantum Yields for Reactions of Bis-(Pentachlorophenyl) Oxalate, Hydrogen Peroxide and Fluorescent Compounds. J. Biolumin. Chemilumin..

[61] Isobe H., Takano Y., Okumura M., Kuramitsu S., Yamaguchi K. (2005). Mechanistic Insights in Charge-Transfer-Induced Luminescence of 1,2-Dioxetanones with a Substituent of Low Oxidation Potential. J. Am. Chem. Soc..

[62] Yue L., Liu Y.-J. (2021). Three S0/S1 Conical Intersections Control Electron-Transfer-Catalyzed Chemiluminescence of 1,2-Dioxetanedione. J. Chem. Theory Comput..

[63] Schaap A.P., Chen T., Handley R.S., DeSilva R., Giri B.P. (1987). Chemical and Enzymatic Triggering of 1,2-Dioxetanes. 2: Fluoride-Induced Chemiluminescence from Tert-Butyldimethylsilyloxy-Substituted Dioxetanes. Tetrahedron Lett..

[64] Schaap A.P., Handley R.S., Giri B.P. (1987). Chemical and Enzymatic Triggering of 1,2-Dioxetanes. 1: Aryl Esterase-Catalyzed Chemiluminescence from a Naphthyl Acetate-Substituted Dioxetane. Tetrahedron Lett..

[65] Lim T., Komoda Y., Nakamura N., Matsunaga T. (1999). Automated Detection of Anti-Double-Stranded DNA Antibody in Systemic Lupus Erythematosus Serum by Flow Immunoassay. Anal. Chem..

[66] Beck S. (1992). Nonradioactive Detection of DNA Using Dioxetane Chemiluminescence. Meth. Enzymol..

[67] Beck S., Köster H. (1990). Applications of Dioxetane Chemiluminescent Probes to Molecular Biology. Anal. Chem..

[68] Bronstein I., Voyta J.C., Murphy O.J., Bresnick L., Kricka L.J. (1992). Improved Chemiluminescent Western Blotting Procedure. Biotechniques.

[69] Baader J.W., Bastos E.L., Berkessel A. (2008). Product Subclass 2: Four-Membered Cyclic Peroxides (1,2-Dioxetanes and 1,2-Dioxetanones). inSci-ence of Synthesis: Houben-Weyl Methods of MolecularTransformations.

[70] Bastos E.L., Leite Ciscato L.F.M., Weiss D., Beckert R., Baader W.J. (2006). Comparison of Convenient Alternative Synthetic Approaches to 4-[(3-Tert-Butyldimethylsilyloxy)Phenyl]-4-Methoxyspiro [1,2-Dioxetane-3, 2′-Adamantane]. Synthesis.

[71] Matsumoto M., Suganuma H., Katao Y., Mutoh H. (1995). Thermal Stability and Chemiluminescence of 3-Alkoxy-3-Aryl-4,4- Diisopropyl-l,2-Dioxetanes. J. Chem. Soc. Chem. Commun..

[72] Matsumoto M., Watanabe N., Kobayashi H., Azami M., Ikawa H. (1997). Synthesis and Chemiluminescence of 3,3-Diisopropyl-4-Methoxy-4-(2-Naphthyl)-1,2-Dioxetanes. Tetrahedron Lett..

[73] Ciscato L.F.M.L., Weiss D., Beckert R., Baader W.J. (2011). Fenchyl Substituted 1,2-Dioxetanes as an Alternative to Adamantyl Derivatives for Bioanalytical Applications. J. Photochem. Photobiol. A Chem..

[74] Watanabe N., Kikuchi M., Maniwa Y., Ijuin H.K., Matsumoto M. (2010). Synthesis of Sulfanyl-, Sulfinyl-, and Sulfonyl-Substituted Bicyclic Dioxetanes and Their Base-Induced Chemiluminescence. J. Org. Chem..

[75] Matsumoto M., Suzuki H., Sano Y., Watanabe N., Ijuin H.K. (2008). Rotamer-Dependent Chemiluminescence in the Intramolecular Charge-Transfer-Induced Decomposition of Bicyclic Dioxetanes Bearing a Hydroxyaryl Group. Tetrahedron Lett..

[76] Watanabe N., Sano Y., Suzuki H., Tanimura M., Ijuin H.K., Matsumoto M. (2010). Synthesis of Thermally Stable Acylamino-Substituted Bicyclic Dioxetanes and Their Base-Induced Chemiluminescent Decomposition. J. Org. Chem..

[77] Matsumoto M., Suzuki H., Watanabe N., Ijuin H.K., Tanaka J., Tanaka C. (2011). Crucial Dependence of Chemiluminescence Efficiency on the Syn/Anti Conformation for Intramolecular Charge-Transfer-Induced Decomposition of Bicyclic Dioxetanes Bearing an Oxidoaryl Group. J. Org. Chem..

[78] Haris U., Kagalwala H.N., Kim Y.L., Lippert A.R. (2021). Seeking Illumination: The Path to Chemiluminescent 1,2-Dioxetanes for Quantitative Measurements and in Vivo Imaging. Acc. Chem. Res..

[79] Kagalwala H.N., Reeves R.T., Lippert A.R. (2022). Chemiluminescent Spiroadamantane-1,2-Dioxetanes: Recent Advances in Molecular Imaging and Biomarker Detection. Curr. Opin. Chem. Biol..

[80] Ciscato L.F.M.L., Bartoloni F.H., Weiss D., Beckert R., Baader W.J. (2010). Experimental Evidence of the Occurrence of Intramolecular Electron Transfer in Catalyzed 1,2-Dioxetane Decomposition. J. Org. Chem..

[81] Ciscato L.F.M.L., Weiss D., Beckert R., Bastos E.L., Bartoloni F.H., Baader W.J. (2011). Chemiluminescence-Based Uphill Energy Conversion. New J. Chem..

[82] Takano Y., Tsunesada T., Isobe H., Yoshioka Y., Yamaguchi K., Saito I. (1999). Theoretical Studies of Decomposition Reactions of Dioxetane, Dioxetanone, and Related Species. CT Induced Luminescence Mechanism Revisited. Bull. Chem. Soc. Jpn..

[83] Adam W., Bronstein I., Trofimov A.V., Vasil’ev R.F. (1999). Solvent-Cage Effect (Viscosity Dependence) as a Diagnostic Probe for the Mechanism of the Intramolecular Chemically Initiated Electron-Exchange Luminescence (CIEEL) Triggered from a Spiroadamantyl-Substituted Dioxetane. J. Am. Chem. Soc..

[84] Adam W., Matsumoto M., Trofimov A.V. (2000). Viscosity Dependence of the Chemically Induced Electron-Exchange Chemiluminescence Triggered from a Bicyclic Dioxetane. J. Am. Chem. Soc..

[85] Adam W., Trofimov A.V. (2000). The Effect of Meta versus Para Substitution on the Efficiency of Chemiexcitation in the Chemically Triggered Electron-Transfer-Initiated Decomposition of Spiroadamantyl Dioxetanes. J. Org. Chem..

[86] Bastos E.L., da Silva S.M., Baader W.J. (2013). Solvent Cage Effects: Basis of a General Mechanism for Efficient Chemiluminescence. J. Org. Chem..

[87] Edwards B., Sparks A., Voyta J.C., Bronstein I. (1990). Unusual Luminescent Properties of Odd- and Even-Substituted Naphthyl-Derivatized Dioxetanes. J. Biolumin. Chemilumin..

[88] Kagalwala H.N., Lippert A.R. (2022). Energy Transfer Chemiluminescent Spiroadamantane 1,2-Dioxetane Probes for Bioanalyte Detection and Imaging. Angew. Chem. Int. Ed..

[89] David M., Jaber Q., Fridman M., Shabat D. (2023). Dual Chemiexcitation by a Unique Dioxetane Scaffold Gated by an OR Logic Set of Triggers. Chem. Eur. J..

[90] Wei X., Huang J., Zhang C., Xu C., Pu K., Zhang Y. (2023). Highly Bright Near-Infrared Chemiluminescent Probes for Cancer Imaging and Laparotomy. Angew. Chem. Int. Ed..

[91] Albrecht H.O. (1928). Über Die Chemiluminescenz des Aminophthalsäurehydrazids. Phys. Chem..

[92] Huang Y., Yue N., Li Y., Han L., Fan A. (2021). One-Step Synthesis of Cationic Gold Nanoclusters with High Catalytic Activity on Luminol Chemiluminescence Reaction. Luminescence.

[93] Li H., Wang J., Du J. (2021). A Novel Luminol Chemiluminescence System Induced by Black Phosphorus Quantum Dots for Cobalt (II) Detection. Talanta.

[94] Chen F., Zhang Y., Li T., Peng D., Qi Z., Song J., Deng T., Liu F. (2023). Discovering Ester and Ether Derivatives of Luminol as Advanced Chemiluminescence Probes. Chin. J. Chem..

[95] Khan P., Idrees D., Moxley M.A., Corbett J.A., Ahmad F., von Figura G., Sly W.S., Waheed A., Hassan M.I. (2014). Luminol-Based Chemiluminescent Signals: Clinical and Non-Clinical Application and Future Uses. Appl. Biochem. Biotechnol..

[96] Radziszewski B.R. (1877). Untersuchungen Über Hydrobenzamid, Amarin Und Lophin. Ber. Der Dtsch. Chem. Ges..

[97] Pavlova E., Kaloyanova S., Deligeorgiev T., Lesev N. (2015). Synthesis and Physicochemical Properties of Novel Lophine Derivatives as Chemiluminescent in Vitro Activators for Detection of Free Radicals. Eur. Biophys. J..

[98] Lopes J.P.B., Câmara V.S., Russowsky D., da Silveira Santos F., Beal R., Nogara P.A., da Rocha J.B.T., Gonçalves P.F.B., Rodembusch F.S., Ceschi M.A. (2018). Lophine and Pyrimidine Based Photoactive Molecular Hybrids. Synthesis, Photophysics, BSA Interaction and DFT Study. New J. Chem..

[99] Melo U., Boaro A., Reis R.A., Silva C.S., Pinto C., Bartoloni F.H. (2021). Evidence for the Formation of 1,2-Dioxetane as a High-Energy Intermediate and Possible Chemiexcitation Pathways in the Chemiluminescence of Lophine Peroxides. J. Org. Chem..

[100] Montano L.A., Ingle J.D. (1979). Investigation of the Lucigenin Chemiluminescence Reaction. Anal. Chem..

[101] Abdussalam A., Chen Y., Yuan F., Ma X., Lou B., Xu G. (2022). Dithiothreitol–Lucigenin Chemiluminescent System for Ultrasensitive Dithiothreitol and Superoxide Dismutase Detection. Anal. Chem..

[102] Shim S., Jeong D.U., Kim H., Kim C.Y., Park H., Jin Y., Kim K.M., Lee H.J., Kim D.H., Bae Y.S. (2022). Discovery of a NADPH Oxidase Inhibitor, (E)-3-Cyclohexyl-5-(4-((2-Hydroxyethyl)(Methyl)Amino)Benzylidene)-1-Methyl-2-Thioxoimidazolidin-4-Oneone, as a Novel Therapeutic for Parkinson’s Disease. Eur. J. Med. Chem..

[103] Chi Q., Chen W., He Z. (2015). Mechanism of Alcohol-Enhanced Lucigenin Chemiluminescence in Alkaline Solution. Luminescence.

[104] Smith K., Ahmed Z., Woodhead J.S., El-Hiti G.A. (2023). Syntheses of Hindered-Polymethylacridinium Esters with Potential for Biological Probe Nanoarchitectonics. J. Oleo Sci..

[105] Smith K., Mu X., Li Z., Holland A.M., Woodhead J.S., El-Hiti G.A. (2023). Synthesis, Structure Elucidation, and Chemiluminescent Activity of New 9-substituted 10-(Ω-(Succinimidyloxycarbonyl)Alkyl)Acridinium Esters. Luminescence.

[106] Ievtukhov V., Zadykowicz B., Blazheyevskiy M.Y., Krzymiński K. (2022). New Luminometric Method for Quantification of Biological Sulfur Nucleophiles with the Participation of 9-cyano-10-methylacridinium Salt. Luminescence.

[107] Roda A., Mirasoli M., Michelini E., Di Fusco M., Zangheri M., Cevenini L., Roda B., Simoni P. (2016). Progress in Chemical Luminescence-Based Biosensors: A Critical Review. Biosens. Bioelectron..

[108] Zhang C., Jin J., Liu K., Ma X., Zhang X. (2021). Carbon Dots-Peroxyoxalate Micelle as a Highly Luminous Chemiluminescence System under Physiological Conditions. Chin. Chem. Lett..

[109] Yahya Kazemi S., Mohammad Abedirad S. (2023). A New Peroxyoxalate Chemiluminescence of Bis (2, 4-Dinitrophenyl) Oxalate (DNPO) Using Pyronin Y as the Fluorophore and Its Application to the Flow-Based Determination of Cysteamine. Spectrochim. Acta A Mol. Biomol. Spectrosc..

[110] Ding Y., Liu W., Wu J., Zheng X., Ge J., Ren H., Zhang W., Lee C.S., Wang P. (2021). Ultrasound-Enhanced Self-Exciting Photodynamic Therapy Based on Hypocrellin B. Chem. Asian J..

[111] Yang Y., Zhang F. (2021). Activatable Chemiluminescent Molecular Probes for Bioimaging and Biosensing. Anal. Sens..

[112] Yang Y., Wang S., Lu L., Zhang Q., Yu P., Fan Y., Zhang F. (2020). NIR-II Chemiluminescence Molecular Sensor for In Vivo High-Contrast Inflammation Imaging. Angew. Chem. Int. Ed..

[113] Digby E.M., Tung M.T., Kagalwala H.N., Ryan L.S., Lippert A.R., Beharry A.A. (2022). Dark Dynamic Therapy: Photosensitization without Light Excitation Using Chemiluminescence Resonance Energy Transfer in a Dioxetane–Erythrosin B Conjugate. ACS Chem. Biol..

[114] Ryan L.S., Gerberich J., Haris U., Nguyen D., Mason R.P., Lippert A.R. (2020). Ratiometric PH Imaging Using a 1,2-Dioxetane Chemiluminescence Resonance Energy Transfer Sensor in Live Animals. ACS Sens..

[115] Hananya N., Press O., Das A., Scomparin A., Satchi-Fainaro R., Sagi I., Shabat D. (2019). Persistent Chemiluminescent Glow of Phenoxy-dioxetane Luminophore Enables Unique CRET-Based Detection of Proteases. Chem. Eur. J..

[116] Park S.Y., Kim M.W., Kang J.-H., Jung H.J., Hwang J.H., Yang S.J., Woo J.K., Jeon Y., Lee H., Yoon Y.S. (2023). Novel NF-ΚB Reporter Mouse for the Non-Invasive Monitoring of Inflammatory Diseases. Sci. Rep..

[117] Yan H., Forward S., Kim K.-H., Wu Y., Hui J., Kashiparekh A., Yun S.-H. (2023). All-Natural-Molecule, Bioluminescent Photodynamic Therapy Results in Complete Tumor Regression and Prevents Metastasis. Biomaterials.

[118] Jiang Y., Shi X., Tang C., Wang F. (2023). Beyond Luciferase-Luciferin System: Modification, Improved Imaging and Biomedical Application. Coord. Chem. Rev..

[119] Yuan M., Fang X., Liu J., Yang K., Xiao S., Yang S., Du W., Song J. (2023). NIR-II Self-Luminous Molecular Probe for In Vivo Inflammation Tracking and Cancer PDT Effect Self-Evaluating. Small.

[120] Nishihara R., Paulmurugan R., Nakajima T., Yamamoto E., Natarajan A., Afjei R., Hiruta Y., Iwasawa N., Nishiyama S., Citterio D. (2019). Highly Bright and Stable NIR-BRET with Blue-Shifted Coelenterazine Derivatives for Deep-Tissue Imaging of Molecular Events in Vivo. Theranostics.

[121] Kim S.-B., Furuta T., Ohmuro-Matsuyama Y., Kitada N., Nishihara R., Maki S.A. Bioluminescent Imaging Systems Boosting Near-Infrared Signals in Mammalian Cells. Photochem. Photobiol. Sci..

[122] Kamiya G., Kitada N., Furuta T., Hirano T., Maki S.A., Kim S.-B. (2023). S-Series Coelenterazine-Driven Combinatorial Bioluminescence Imaging Systems for Mammalian Cells. Int. J. Mol. Sci..

[123] Gomollón-Bel F. (2021). IUPAC Top Ten Emerging Technologies in Chemistry 2021. Chem. Int..

[124] Cao J., Lopez R., Thacker J.M., Moon J.Y., Jiang C., Morris S.N.S., Bauer J.H., Tao P., Mason R.P., Lippert A.R. (2015). Chemiluminescent Probes for Imaging H2S in Living Animals. Chem. Sci..

[125] Cao J., Campbell J., Liu L., Mason R.P., Lippert A.R. (2016). In Vivo Chemiluminescent Imaging Agents for Nitroreductase and Tissue Oxygenation. Anal. Chem..

[126] Green O., Eilon T., Hananya N., Gutkin S., Bauer C.R., Shabat D. (2017). Opening a Gateway for Chemiluminescence Cell Imaging: Distinctive Methodology for Design of Bright Chemiluminescent Dioxetane Probes. ACS Cent. Sci..

[127] Roth-Konforti M.E., Bauer C.R., Shabat D. (2017). Unprecedented Sensitivity in a Probe for Monitoring Cathepsin B: Chemiluminescence Microscopy Cell-Imaging of a Natively Expressed Enzyme. Angew. Chem. Int. Ed..

[128] Shelef O., Sedgwick A.C., Pozzi S., Green O., Satchi-Fainaro R., Shabat D., Sessler J.L. (2021). Turn on Chemiluminescence-Based Probes for Monitoring Tyrosinase Activity in Conjunction with Biological Thiols. Chem. Commun..

[129] Bruemmer K.J., Green O., Su T.A., Shabat D., Chang C.J. (2018). Chemiluminescent Probes for Activity-Based Sensing of Formaldehyde Released from Folate Degradation in Living Mice Kevin. Angew. Chem. Int. Ed..

[130] Ye S., Yang B., Wu M., Chen Z., Shen J., Shabat D., Yang D. (2022). Recurring Real-Time Monitoring of Inflammations in Living Mice with a Chemiluminescent Probe for Hypochlorous Acid. CCS Chem..

[131] Hananya N., Eldar Boock A., Bauer C.R., Satchi-Fainaro R., Shabat D. (2016). Remarkable Enhancement of Chemiluminescent Signal by Dioxetane-Fluorophore Conjugates: Turn-ON Chemiluminescence Probes with Color Modulation for Sensing and Imaging. J. Am. Chem. Soc..

[132] Kagalwala H.N., Gerberich J., Smith C.J., Mason R.P., Lippert A.R. (2022). Chemiluminescent 1,2-Dioxetane Iridium Complexes for Near-Infrared Oxygen Sensing. Angew. Chem. Int. Ed..

[133] Reeves A.G., Subbarao M., Lippert A.R. (2017). Imaging Acetaldehyde Formation during Ethanol Metabolism in Living Cells Using a Hydrazinyl Naphthalimide Fluorescent Probe. Anal. Methods.

[134] Cao J., An W., Reeves A.G., Lippert A.R. (2018). A Chemiluminescent Probe for Cellular Peroxynitrite Using a Self-Immolative Oxidative Decarbonylation Reaction. Chem. Sci..

[135] An W., Ryan L.S., Reeves A.G., Bruemmer K.J., Mouhaffel L., Gerberich J.L., Winters A., Mason R.P., Lippert A.R. (2019). A Chemiluminescent Probe for HNO Quantification and Real-Time Monitoring in Living Cells. Angew. Chem. Int. Ed..

[136] Ryan L.S., Gerberich J., Cao J., An W., Jenkins B.A., Mason R.P., Lippert A.R. (2019). Kinetics-Based Measurement of Hypoxia in Living Cells and Animals Using an Acetoxymethyl Ester Chemiluminescent Probe. ACS Sens..

[137] Quimbar M.E., Davis S.Q., Al-Farra S.T., Hayes A., Jovic V., Masuda M., Lippert A.R. (2020). Chemiluminescent Measurement of Hydrogen Peroxide in the Exhaled Breath Condensate of Healthy and Asthmatic Adults. Anal. Chem..

[138] Calabria D., Guardigli M., Mirasoli M., Punzo A., Porru E., Zangheri M., Simoni P., Pagnotta E., Ugolini L., Lazzeri L. (2020). Selective Chemiluminescent TURN-ON Quantitative Bioassay and Imaging of Intracellular Hydrogen Peroxide in Human Living Cells. Anal. Biochem..

